# Surface water monitoring of chemicals associated with animal husbandry in an agricultural region in the Netherlands using passive sampling

**DOI:** 10.1007/s10661-024-12818-5

**Published:** 2024-06-28

**Authors:** Nikola Rakonjac, Erwin Roex, Henry Beeltje

**Affiliations:** 1 Soil Physics and Land Management Group, Wageningen University, Droevendaalsesteeg 3, 6708PB Wageningen, the Netherlands; 2 National Institute for Public Health and the Environment (RIVM), Bilthoven, the Netherlands; 3 AQUON, Tiel, the Netherlands

**Keywords:** Surface water, Antibiotics, Antiparasitics, Hormones, Disinfectants, Passive sampling

## Abstract

Compounds originating from animal husbandry can pollute surface water through the application of manure to soil. Typically, grab sampling is employed to detect these residues, which only provides information on the concentration at the time of sampling. To better understand the emission patterns of these compounds, we utilized passive samplers in surface water to collect data at eight locations in a Dutch agricultural region, during different time intervals. As a passive sampler, we chose the integrative-based Speedisk® hydrophilic DVB. In total, we targeted 46 compounds, among which 25 antibiotics, three hormones, nine antiparasitics, and nine disinfectants. From these 46 compounds, 22 compounds accumulated in passive samplers in amounts above the limit of quantification in at least one sampling location. Over the 12-week deployment period, a time integrative uptake pattern was identified in 53% of the examined cases, with the remaining 47% not displaying this behavior. The occurrences without this behavior were primarily associated with specific location, particularly the most upstream location, or specific compounds. Our findings suggest that the proposed use of passive samplers, when compared in this limited context to traditional grab sampling, may provide enhanced efficiency and potentially enable the detection of a wider array of compounds. In fact, a number of compounds originating from animal husbandry activities were quantified for the first time in Dutch surface waters, such as flubendazole, florfenicol, and tilmicosine. The set-up of the sampling campaign also allowed to distinguish between different pollution levels during sampling intervals on the same location. This aspect gains particular significance when considering the utilization of different compounds on various occasions, hence, it has the potential to strengthen ongoing monitoring and mitigation efforts.

**Supplementary Information:**

The online version contains supplementary material available at 10.1007/s10661-024-12818-5.

## Introduction

Veterinary pharmaceuticals (VPs) are compounds used to treat or prevent diseases of animals. Depending on the compound, a certain fraction is excreted unchanged via urine and feces and ends up in the animal manure after administration (Berendsen et al., 2018). In the Netherlands, the use of VPs (especially antibiotics) in livestock farming has been drastically reduced over the last decade but used quantities are still significant (De Greeff et al., 2021). In addition, the Dutch livestock farming sector produces considerable amounts of manure and consequently VP residues frequently end up on the soil via the application of manure as fertilizer on arable land (Lahr et al., 2018; Rakonjac et al., 2022; Rakonjac, 2023; Rakonjac et al.. 2023). Depending on the physico-chemical characteristics of the compound and the soil properties, a portion of these residues may further reach the surface water via flow paths (e.g., surface runoff), potentially resulting in the pollution of surface water systems over long distances downstream of the application areas (Bailey et al., 2015).

Next to frequently used VPs, surface water in the Netherlands may also be exposed to other classes of compounds associated with animal husbandry, from which naturally occurring hormones and disinfectants are the most prominent. An extensive Dutch study (Vethaak et al., 2005) has shown that in streams in agricultural areas in the Netherlands, relatively high levels of naturally occurring hormones appear. The same study also showed that these compounds can have detrimental effects on organisms. The class of Quaternary Ammonium Compounds (QACs) belongs to the disinfectants that have the potential to come into contact with Dutch surface water. These compounds are known to have a broad range of applications (Mulder et al., 2018) and have recently been identified in manure, forage, and agricultural soil samples in the Netherlands (Buijs et al., 2019). The occurrence of these compounds in surface water within agricultural regions of the Netherlands has not been made public thus far, regardless of their potential to pose a risk to human health upon exposure (Hrubec et al., 2021).

Despite the considerable amount of data confirming the global prevalence of the aforementioned compounds, particularly VPs, in aquatic systems Umwelt Bundesamt (2022), and some studies suggesting potential risks to the aquatic environment (Kemper, 2008; Kools et al., 2008), the evidence on their occurrence in surface waters remains limited and scarce. This is especially the case for smaller streams that are not directly used as a source for drinking water but rather for agricultural activities (e.g., irrigation). Besides, these small streams act as breeding grounds for numerous aquatic species, and disturbance of their habitat by VPs may have eventual severe effect on aquatic ecosystems. For this reason, the monitoring of VPs in surface water has been highlighted by the European Commission Strategic Approach to Pharmaceuticals in the Environment (2019) as one of the research questions which remains to be tackled.

In aquatic systems, monitoring of compounds is typically based on grab sampling of the aqueous phase, followed by chemical analysis of the targeted compounds. Although grab sampling is a widely used and accepted method (e.g., in European frameworks), it only provides a snapshot of the concentration at the moment of sampling and may not account for temporal fluctuations due to irregular emission patterns or variations in water flow. This is especially relevant for chemicals with diffuse environmental pathways, such as compounds associated with manure application. To improve sampling sensitivity and integrate temporal changes, time-integrative passive sampling has been proposed (Górecki & Namieśnik, 2002; Vrana et al., 2005). This method involves deploying an adsorption or absorption material in the aquatic environment for a certain time period, allowing for accumulation of compounds and monitoring of low aqueous concentrations. During deployment, the freely dissolved fraction of contaminants accumulate into the sampler material at a rate controlled by their diffusion through the water boundary layer (WBL) at the surface of the sampler. When the flow rate is higher, the WBL will be thinner and uptake will therefore be faster.

Passive samplers can be broadly categorized into two types: equilibrium-based samplers and integrative-based samplers (Vrana et al., 2009). Equilibrium-based samplers mainly absorb hydrophobic substances from the aqueous phase because of their higher affinity for the sampler material (mostly polymeric) compared to the aqueous phase. Based on the sampler-water partition coefficient (Kpw) of a compound, the Sampling Rate (Rs) per compound can be determined and consequently the aqueous concentration. Integrative-based samplers bind the substances at the surface of the adsorption material, even if only a part of the molecule has the appropriate affinity. Assuming that sufficient binding material is present in the sampler, integrative-based samplers are supposed not to reach equilibrium and display compound uptake that is linear over time. Especially for substances with polar functional groups that avoid absorption by equilibrium-based samplers, like VPs, integrative-based sampler has proven to be very suitable, particularly for the purpose of qualitative screening (Gong et al., 2018; Moschet et al., 2015; Škodová et al., 2016). Although attempts have been made (Hamers et al., 2018), sampling rates for integrative-based samplers are not yet robust enough to supply reliable aqueous concentrations (Harman et al., 2012). Therefore, we decided to base our findings on the amounts per sampler.

The most comprehensive study to date in the Netherlands shows that only a limited number of VPs could be detected in surface water located in an agricultural area, which according to the authors could be partly attributed to the low monitoring frequency (Lahr et al., 2018). This was confirmed by a recent overview (Lahr et al., 2019), in which in fact passive sampling was mentioned as an alternative method to overcome this problem. The lack of information on these chemicals in Dutch surface waters despite significant use justifies further investigation and passive samplers offer a potential way to do this. Given our primary focus on VPs, we selected the integrative-based Speedisk as our preferred passive sampler. Earlier studies have already shown the robustness of the Speedisk as a reliable choice for environmental monitoring of various chemical compounds (De Weert et al., 2020; Zillien et al., 2019, 2024). We further discussed the performance of passive samplers, spatial and temporal trends, and the amount of compounds accumulated on the samplers, if detected. In this study, we intended to implement a screening approach aimed at identifying the array of compounds present in the agricultural surface water, without an attempt to estimate their concentrations due to the limitations associated with the used methodology.

## Materials and methods

### Sampling locations and deployment scheme

**Fig. 1:**
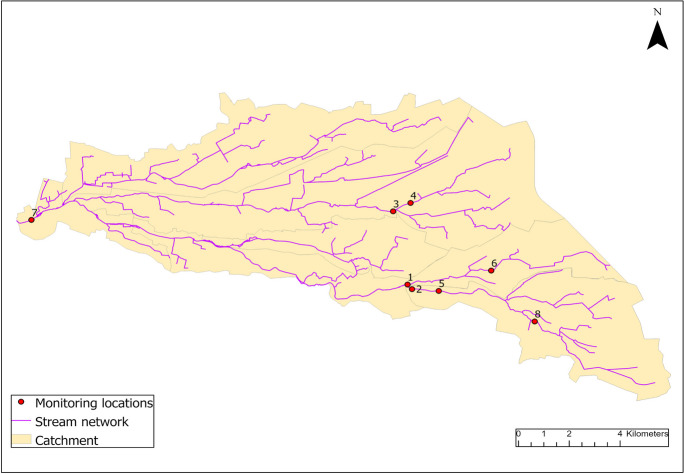
Selected agricultural region in the middle of the Netherlands with eight monitoring locations. Water flow is in the direction from east to west, where location 7 represents the catchment outlet

The study area is an agricultural region in the middle of the Netherlands. A number of locations were visited together with a representative of the regional water authority in February 2020 and assessed in terms of their suitability as potential monitoring sites. The criteria considered during those visits were as follows: the extent of livestock breeding, livestock sector, potential inputs of chemicals, manure application rates, soil and hydrological characteristics, previous VPs measurements in surface water (if available), and feasibility of passive samplers deployment. Preference was given to areas where many cow and/or pig farms were located on sandy soil, where fields (i.e., manure application areas) were located nearby water streams, and where contribution of human pharmaceuticals was minimized (e.g., without upstream discharges from WWTP). Eight sampling locations were selected using these criteria, as illustrated in Fig. 1.

The total catchment area, and therefore the total area draining into the sampling location number 7, is around 158 km^2^. This area is a valley formed in the Saale period (150 ka BC) and its shallow subsurface mostly consists of sandy deposits from peri-glacial and coastal origin. In fact, around 75% of all fields on which manure (i.e., VPs) is applied in this area are on sandy soils, while the rest are typically on peat (11%) and clay soils (8%). The most dominant crop in this area is maize covering almost 80% of the fields, followed by grasslands with around 15% (Kros et al., 2019).

In order to capture the most prominent annual VP concentration peaks in surface water, the local manure application patterns were assessed. Since the majority of agricultural fields in the area have a combination of sandy soil and maize, that situation was assumed as a representative for the whole area. In that case, more than 95% of yearly (slurry) manure loads are applied to the soil between March and June (De Vries et al., 2023; Kros et al., 2019). This practice might slightly deviate between farms but is in accordance with the Dutch national regulations for manure application (RVO, 2022). Consequently, as the most suitable timeframe for sampling, the period between 25th March and 17th June was chosen. In total, we targeted 46 compounds, among which 25 antibiotics, three hormones, nine antiparasitics, and nine disinfectants. The list of all targeted compounds is given in Supplementary Material (see SM1).

**Fig. 2:**
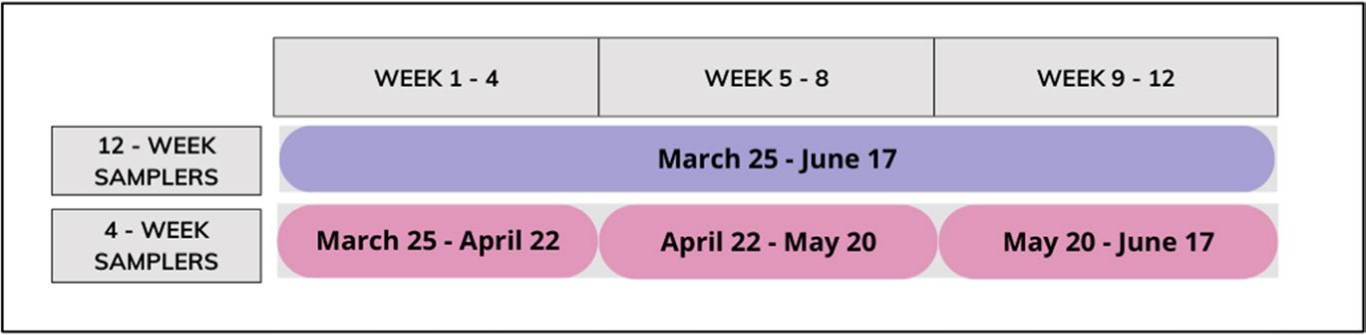
Sampling timeline for eight investigated locations

The sampling strategy was based on deploying two pairs of passive samplers at each location. A first set of samplers was deployed for 12 consecutive weeks. Further, three different sets of samplers were deployed, each for 4 consecutive weeks during the time period of the first set, for two different reasons. First, with this set-up, we were able to evaluate the time integrative sampling of the passive samplers, similar to the study by De Weert et al. (2020). Second, the analysis of the samplers which were deployed for 4 weeks enabled us to investigate whether variability in accumulated amounts within the 12 weeks occurred. The samplers were deployed in time series following the timeline outlined in Fig. 2.

### Passive sampler preparation

In this study, the integrative-based Speedisk (BAKERBOND® Speedisk®) was chosen as a passive sampler. The Speedisk consists of a polypropylene container filled with ~ 600 mg of hydrophilic DiVinylBenzene (DVB) sorbent placed on the bottom as a uniform layer covered with a fine nylon mesh and a 0.5 mm glass fiber filter held in place by a 2 mm nylon mesh. The preparation of passive samplers was done by following a similar procedure as described in Hamers et al. (2018) and De Weert et al. (2020); hence, we refer to those studies for more details. Per sampling site and sampling occasion, two Speedisks were deployed and considered one sampler, of which the combined extracts were used for chemical analysis. At the end of dedicated sampling periods, samplers were pulled out and cleaned with local surface water and then stored in jars at − 20° C prior to extraction. During deployment, visual inspections of the samplers were performed on a regular basis, and when needed, plant material and debris were removed.

### Extraction and processing of the samples

Before extraction, the Speedisks were spiked directly on the surface with analytical recovery internal standards consisting of certified mass-labeled compounds representing the selected compounds to be analyzed, see SM6 for detailed information on the compounds selected. Extraction of the Speedisks was performed according to the Dutch protocol (NEN 2022). This procedure relies on a number of steps, in order to extract as much as possible compounds from the samplers. First, the analytes were extracted from samplers with 2 mL methanol (Biosolve, ULC/MS), followed by an extraction with five times 5 mL dichloromethane (Merck, Supelco EMSURE). These eluates were combined and, after drying with sodium sulfate (Merck ACS reagent) over a glass frit, concentrated to 2–3 mL. In the second step, an extraction with four times 5 mL methanol was performed, which was added to the eluate from the first step. This combined extract was further concentrated under a stream of nitrogen to a volume of approximately 0.9 mL, and methanol was added to reach a volume of 1 mL. In the third step, the analytes were extracted with four times 5 mL 1% formic acid (FA ≥ 99% (Biosolve, ULC/MS)) methanol. This extract was completely dried under a stream of nitrogen, and again methanol was added to reach a volume of 1 mL. All extractions were performed by using a vacuum system (JT Baker®). Before chemical analysis, 100 µL of the methanol and 100 µL of the FA extract were combined and analyzed. Procedure blanks were obtained by extracting two unexposed dry samplers, which followed the same procedure as the exposed samplers. These were also included in the subsequent analyses.

In a parallel spike and recovery experiment, the recovery of all the substances on the passive sampler was tested in which a standard mixture of all the tested compounds in 1 L was run over a Speedisk (*n* = 8). Afterwards, the compounds were eluated similarly to the field samplers, and the concentration in the eluate was compared with the concentration in the original mixture. Average analytical recoveries are given in SM2. All extracts were stored in a dark environment at − 20 °C until further analysis.

### Chemical analysis

The final extracts were analyzed by an Agilent 1260 series high-performance liquid chromatography coupled with an Agilent 6460 triple quadrupole LC/MS with Jetstream Electron Spray Ionization (ESI) and multiple reaction monitoring (MRM). A sample volume of 5 µL was injected with a flow rate of 0.5 mL min^−1^. Depending on the target compounds, different columns and methods were used. VPs were separated with a Gemini 3 µm NX-C18 110 Å (100 × 2 mm) column with a gradient of 1 mM ammonium fluoride in 100% Milli-Q water (eluent A) and 100% methanol (eluent B). The hormones were separated with a Gemini 3 µm NX-C18 110 Å (100 × 2 mm) column using a gradient of 100% Milli-Q water (eluent A) and 100% methanol (eluent B), and the disinfectants were separated with a Kinetex 2.6 µm Biphenyl 100 Å (100 × 2.1 mm) column, using a gradient of 100% MQ with 0.002% Formic Acid (eluent A) and 100% MeOH with 0.002% Formic Acid (eluate B). The target compounds were determined with one precursor ion and two product ions. For detailed information about mass-to-charge ratios, retention times and ratios see SM4. For quantification of the target compounds, external calibration was performed with known amounts of the analytes in 9 steps with concentrations ranging between 0 and 810 ng mL^−1^, depending on the compound. The limit of detection (LOD) and limit of quantification (LOQ) of the analytes were determined with signal-to-noise ratios of 1:3 and 1:10, respectively. The method resulted in values for LOQ ranging between 3 and 60 ng mL^−1^. The results were expressed as ng/sampler, where a sampler consists of 2 Speedisks which were deployed, extracted, and analyzed at the same time.

## Results

### Total amount per sampler

From the 46 targeted compounds, 22 accumulated in passive samplers in amounts above the LOQ in at least one sampling period in one of the eight locations. These compounds are shown in Table 1 and are further addressed in this paper. The remaining compounds (24) may have been present in streams in amounts < LOQ or undetected due to low recovery. Low recovery could in principle be caused by one or a combination of at least three processes, namely poor uptake by the Speedisk, poor extraction from the Speedisk, or loss of analytes during the sample preparation. The extraction procedure used in this study is more extensive than regular, with the aim to extract as much as possible compounds from the Speedisk. Unpublished results from our labs have shown that the loss of analytes during sample preparation does not occur. Therefore the low recovery is most probably predominantly caused by the poor uptake by the Speedisk. This is particularly evident for the group of tetracyclines, for which all recoveries were below 40% (see SM2). Although the group of tetracyclines displays a high hydrophilicity, other hydrophilic compounds which were targeted showed a high recovery. Most probably it is the high hydrophilicity of the tetracyclines, combined with specific molecular structures present in this group of compounds which causes the low affinity for the Speedisk. The measured amounts of all the detected compounds in the Speedisk extracts, together with LOD and LOQ values, are given in the SM1.

**Table 1:** Detected compounds, compound type, number of detected locations, and range of detected quantities (12-week samplers)

Compound	Type	Cas no	No. of detected locations	Detected quantities [ng/sampler]^a^
Florfenicol	Antibiotic	73231–34-2	8/8	15–70.3
Flumequine	Antibiotic	42835–25-6	8/8	6.3–13.3
Lincomycin	Antibiotic	154–21-2	6/8	1.3^b^–3.3
Oxytetracycline	Antibiotic	79–57-2	3/8	64.5–142
Sulfadiazine	Antibiotic	68–35-9	8/8	29–547
Sulfamethazine	Antibiotic	57–68-1	8/8	35.2–139
Sulfamethoxazole	Antibiotic	723–46-6	8/8	1.5^b^–46.3
Sulfamethoxypyridazine	Antibiotic	80–35-3	8/8	1.9^b^–6
Sulfapyridine	Antibiotic	144–83-2	5/8	1.3^b^–13
Tilmicosine	Antibiotic	108050–54-0	8/8	4.4–74
Trimethoprim	Antibiotic	738–70-5	7/8	1.1^b^–18
Tylosin	Antibiotic	1401–69-0	4/8	3–6.2
Flubendazole	Antiparasitic	31430–15-6	8/8	3.4–18.5
Mebendazole	Antiparasitic	31431–39-7	1/8	3.3^c^
Permethrin	Antiparasitic	52645–53-1	1/8	3.0^c^
Fipronil sulfone	Metabolite	120068–36-2	7/8	1.2^b^–5.4
Estrone	Hormone	53–16-7	8/8	3–14.2
Benzyldimethyl-dodecylammonium chloride (BAC-C12)	Biocide	139–07-1	8/8	11–33
Benzyldimethyl-tetradecylammonium chloride (BAC-C14)	Biocide	139–08-2	8/8	10–24
Benzyldimethyl-hexadecylammonium chloride (BAC-C16)	Biocide	122–18-9	8/8	2.1^b^–6.4
Benzyldimethyl-octadecylammonium chloride (BAC-C18)	Biocide	122–19-0	2/8	2.2^b^
Didecyldimethyl-ammonium chloride (DDAC-C10)	Biocide	7173–51-5	8/8	6.6–76

^a^The range of detected amounts accumulated in passive samplers expressed in ng per sampler is defined by the lower border, which represents the lowest quantity (above LOD) found at one of the locations on a 12-week sampler, and the higher border, which represents the highest quantity found at one of the locations on a 12-week sampler. For the amounts on 4-week samplers, see SM1

^b^Value above the LOD but below the LOQ

^c^Incidental occurrence—compound detected only at one location during one of the sampling intervals

Among the 22 compounds accumulated above the LOQ, two of them were found to be insufficiently well taken up by the Speedisk which was expressed by the low recovery rate (< 70%) in the parallel lab experiment. The mentioned compounds are oxytetracycline and tylosin, and those were therefore discarded from further consideration. Furthermore, mebendazole and permethrin were only incidentally detected, occurring at a single location and during a single time interval. Consequently, these compounds were also excluded from subsequent investigation. A comprehensive analysis was conducted to evaluate the uptake of the remaining 18 compounds across 8 locations, resulting in a total of 144 location-compound combinations. Out of these combinations, 36 featured compounds that were either not detected or were found at levels below the LOQ, which applied to both the 12- and 4-week samplers. Notable instances of the latter scenario included fipronil sulfone, lincomycin, and BAC-C18. As a result, 108 location-compound combinations remained for further examination.

To further investigate the uptake and potential influence of specific location circumstances or compound characteristics on the uptake process, we introduced a metric termed the average Time-Integrative Uptake Coefficient (TUC). The TUC was computed as the sum of 4-week amounts divided with the 12-week amounts for each location and compound. This procedure resulted in the exclusion of 9 combinations where a compound was detected in the 12-week samplers but not in any of the 4-week samplers, leading to a total of 99 TUC values (Table SM3).

Using this indicator, we first categorized the uptake behavior into three distinct groups: time integrative uptake, potentially time integrative uptake, and non-integrative uptake over time. Time integrative uptake was characterized by TUC values falling within the range of 0.5 to 1.5, where a TUC value of 1 indicated perfect time integrative uptake. In some instances, although a compound was not detected during all four sampling periods, the accumulated amounts above the LOQ still suggested that the samplers potentially exhibited time integrative uptake (see Fig. 6). Consequently, these scenarios were classified into the second defined category. The third category included situations where TUC values fell outside the previously mentioned range, encompassing scenarios where data were available for all four sampling periods as well as instances where data were incomplete. In cases where complete data for all periods were not available and based on the accumulated amounts above LOQ, no indications of time integrative uptake at a specific location could be discerned. In total, 53% of the combinations implied time integrative uptake (first and second category), while the rest (47%) showed non-integrative uptake in time.

Upon further investigation of the TUC values, we noted that the majority of compounds at location 8 exhibited deviations from the time integrative uptake pattern, featuring TUC values significantly exceeding 1, specifically with an average value of 4.18 (*n* = 13). A closer look at the results of the individual samplers of location 8 showed that especially in the first 4 weeks, the concentrations on the samplers for most of the compounds detected were already higher than the concentration after 12 weeks (see also SM1). It has been shown that the uptake rate of compounds by several types of passive samplers can be substantially influenced by hydrological conditions (i.e., water flow). For instance, both Shi et al. (2014) and Guibal et al. (2020) showed that uptake rates positively correlate with flow rates. This could offer a reasonable explanation for the distinct uptake patterns observed at location 8. This particular location is positioned furthest upstream (see Fig. 1), featuring the lowest flow rate. However, it is also susceptible to significant fluctuations during the spring season due to rainfall events. Besides, during some of the visual inspections, it was observed that especially at this location, the samplers were overgrown with different macrophytes and algae, a process which has been suggested to be an issue for passive samplers suitable for sampling polar compounds (Harman et al., 2012). Finally, it could also be argued that the amount of the binding material within the Speedisk might not have been sufficient enough, thereby hampering a time integrative uptake during the whole period. However, based on indicative uptake rates, the DVB mass per Speedisk and the partition coefficient between DVB and water, De Weert et al. (2020) calculated a linear uptake time window for the Speedisk sampler from 128 days under very turbulent water-flow conditions to 1935 days under almost static conditions. As these calculated linear uptake windows exceed the maximum sampling period of 84 days in the present study, the Speedisk is supposed to remain in the linear uptake phase during deployment. As no aqueous concentrations were determined during the deployment period of the Speedisks, a definite reason for the non-integrative uptake process, especially at location 8 cannot be given. It is possibly a combination of the processes mentioned above, combined with possible fluctuating aqueous concentrations, which may have result in highly fluctuating amounts taken up by the samplers in the consecutive time intervals.

Next to location 8, one specific group of compounds showed TUC values which substantially differed from time integrative uptake, namely the QACs (BAC-C12, BAC-C14, BAC-C16, BAC-C18, DDAC-C10) (see Table SM3). This could be caused by the lower affinity of cationic compounds, like QACs, to passive samplers, which has been reported before (Moschet et al., 2015). Although the QACs showed a high affinity to the Speedisk in the (short term) lab experiments (see SM2), this lower affinity might have been more pronounced during longer deployment periods, when compounds with a lower affinity may have to compete with compounds with a higher affinity to the Speedisk. This phenomenon seems to be the most pronounced at location 1, where high concentrations of a number of QACs were detected in the samplers deployed for 4 weeks, probably caused by a peak moment. These high concentrations were not reflected by the passive samplers which were deployed for 12 weeks (SM1). In those cases, the results from the Speedisk do not reflect the chemical constitution of the total deployment period, which would make these samplers less suitable for these groups of compounds. It is therefore recommended to investigate the affinity of QACs to Speedisks in additional lab experiments.

**Fig. 3:**
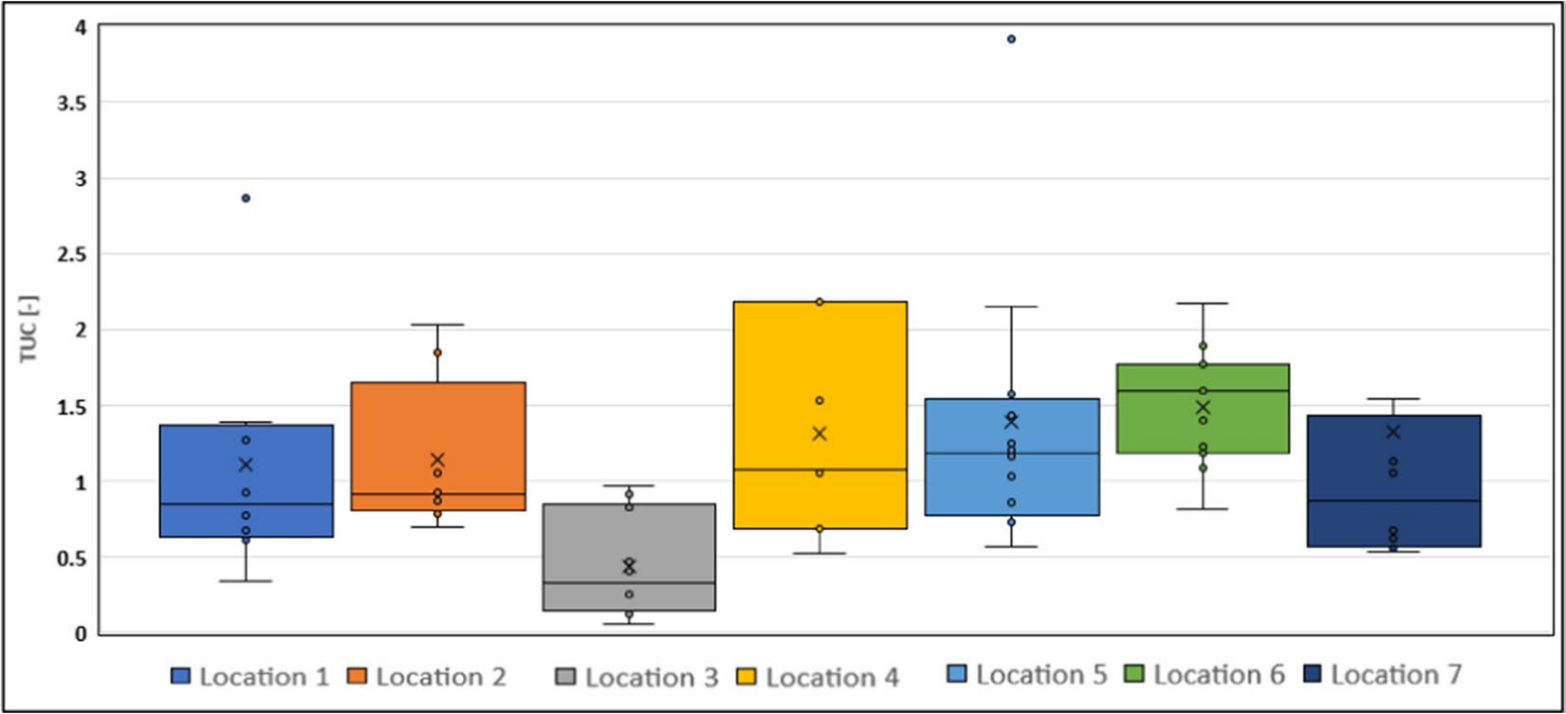
Variation in TUC values across 7 locations—Box and Whisker Plot representation, indicating the minimum value, first quartile (the bottom line of the box), median (the line inside the box), mean (x in the box), third quartile (the top line of the box) and maximum value of a data set. The number of dots depends on the available TUC values for specific location, as indicated in the SM3

In fact, upon exclusion of combinations associated with location 8 and with QACs, the average TUC values per location and compound exhibit a much closer alignment with the ideal TUC value of 1 in 80% of the cases. The distribution of TUC values in this context is visually presented in Fig. 3, with a detailed compilation of values provided in SM3.

**Fig. 4:**
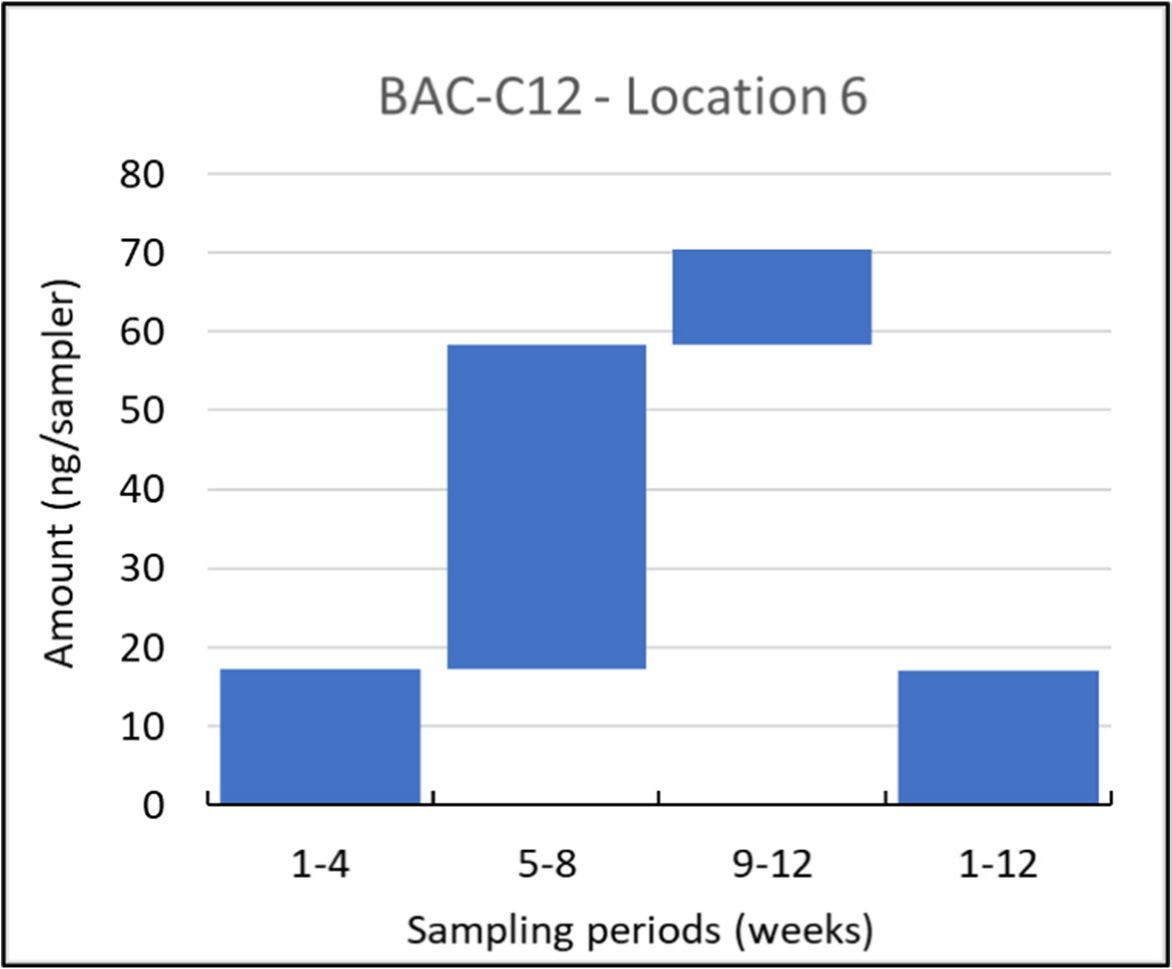
Uptake of BAC-C12 in consecutively and in parallelly exposed Speedisk samplers at location 6, shown as the amounts on the samplers (ng/sampler) sampled during the weeks indicated at the *x*-axis. This example is considered a representative of the non-integrative uptake in time

**Fig. 5:**
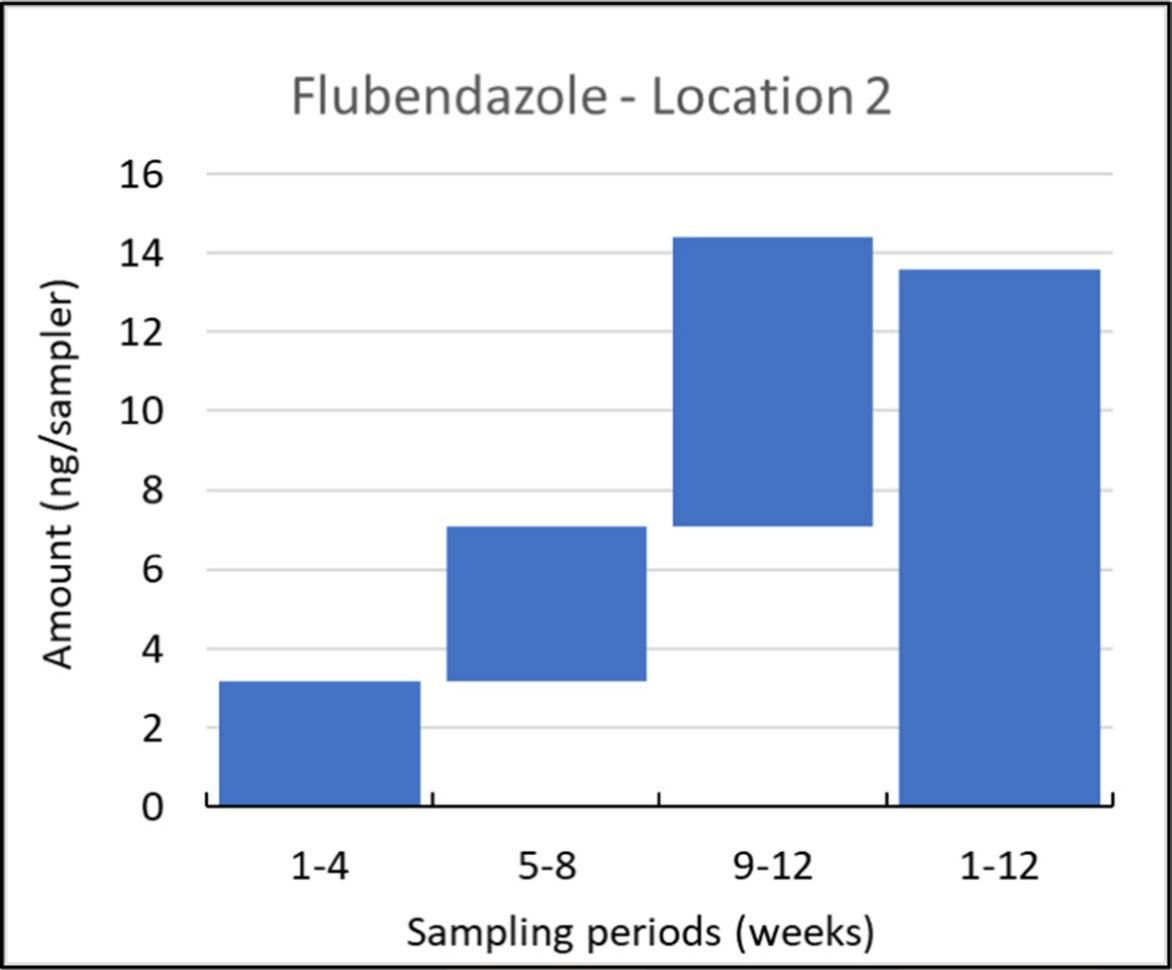
Uptake of flubendazole in consecutively and in parallelly exposed Speedisk samplers at location 2, shown as the amounts on the samplers (ng/sampler) sampled during the weeks indicated at the *x*-axis. This example is considered a representative of the time integrative sampler uptake

Among the 13 compounds detected at all sampling locations (as listed in Table 1), eight exhibited consistent detection throughout all four sampling periods. These compounds are estrone, florfenicol, flubendazole, flumequine, sulfadiazine, sulfamethazine, BAC-C12, and BAC-C14. Notably, BAC-C12 and BAC-C14 showcased distinct non-integrative uptake patterns, as depicted for BAC-C12 in Fig. 4. On the other hand, florfenicol, flubendazole, and flumequine displayed good time integrative sampling properties across most locations, as depicted in the example of flubendazole in Fig. 5.

**Fig. 6:**
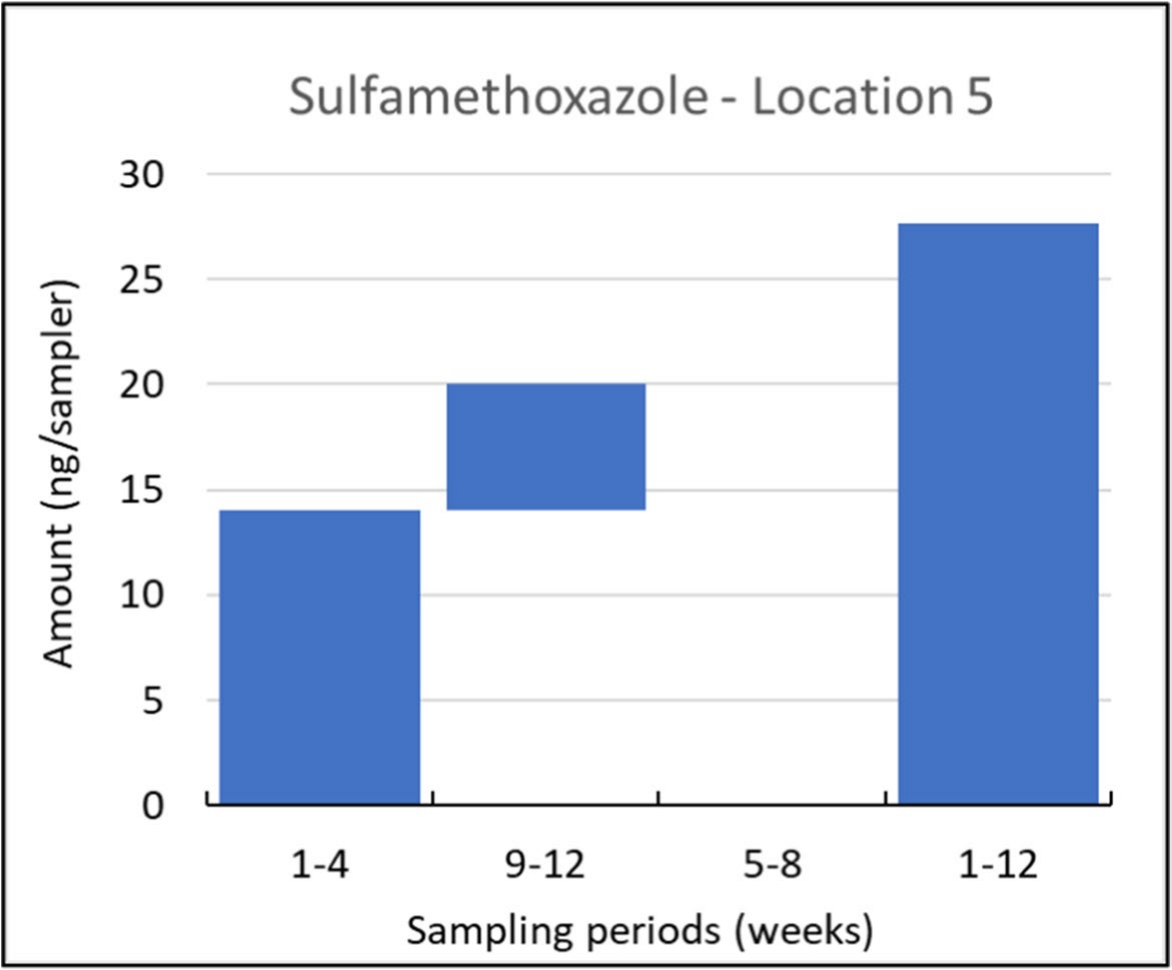
Uptake of sulfamethoxazole in consecutively and in parallelly exposed Speedisk samplers at locations 5, shown as the amounts on the samplers (ng/sampler) sampled during the weeks indicated at the *x*-axis. This example illustrates the category of possible time-integrative uptake

Among the compounds detected at all locations but not consistently throughout all sampling periods, sulfamethoxazole can be considered an exemplar of the possibly time integrative uptake category, as previously defined. At location 5, this compound was not detected with 5–8 week samplers but was found during all other time intervals, as illustrated in Fig. 6. This effect can be caused by fluctuating concentrations of a compound in the water resulting in fluctuating uptake during the different intervals. This immediately shows the added value of the different sampling intervals; the deployment of 12 weeks produces more often a result > LOQ than the deployment period of 4 weeks, as the deployment time of 4 weeks is sometimes not long enough to reach an amount in the sampler > LOQ. On the other hand, the results of the samplers which are deployed for 4 weeks give detailed information concerning the different time periods within these 12 weeks.

Generally, deviations from time integrative sampling patterns could potentially arise due to measurement errors in quantities near the limit of detection. An alternative explanation is that the Speedisk might demonstrate a quicker uptake for specific compounds during the initial stages of deployment, aligning with observations in the studies by Mazzella et al. (2007) and Bernard et al. (2022). This eventually results in a higher sum uptake for the 1–4, 5–8, and 9–12 weeks deployment than for the Speedisk constantly deployed in parallel (De Weert et al., 2020).

### Spatial trends

From the 22 compounds found above LOQ in the investigated area, the highest number of compounds detected on the samplers during one sampling period was 19 at locations 3 and 5. At location 3, those were detected on the 12-week samplers, while at location 5 those were detected both on 12-week and 1–4-week samplers. The lowest number of detected compounds (11) was at location 4. Even though locations 3 and 4 are situated relatively close to each other (location 3 is around 700 m downstream from location 4), the number of detected compounds was quite different. All the compounds which were found in location 4 were also found in location 3, meaning that location 4 had a substantial influence on location 3. But next to that, another 8 compounds were detected on location 3, which indicates most probably the influence of an additional stream which fed location 3 from the south-east side of the catchment (see Fig. 1).

In addition, we compared the 12-week samplers at locations 2 and 5 (upstream of 2), which were fed by the approximately same catchment area. Out of the 19 compounds detected at location 5, 17 were also detected at location 2, with the remaining two compounds being close to the LOQ at location 5. No additional compounds were found at location 2. Furthermore, the compounds detected both at locations 2 and 5 were also found at location 8, which is located far upstream, indicating that the stream network in that part of the catchment is consistently exposed to the same set of compounds. The same conclusion could be drawn for locations 6 and 1, as the same set of compounds was detected at these locations using both 12- and 4-week samplers. All the compounds found at location 7 were also detected somewhere upstream, typically in both investigated catchment parts. This finding indicates that a monitoring location like location 7 may serve as a surveillance monitoring location, providing an assessment of the overall status of the upstream water bodies. The other locations located further upstream are able to provide more detailed information about possible sources of compounds detected.

It is important to note that the precipitation which feeds into to a stream network can have diverse effects on fluxes of compounds in this network, substantially influenced by the physical–chemical properties of the compound. This may eventually also affect the accumulated amounts on samplers in different ways. On one hand, dilution of the compound might occur, decreasing the aqueous concentration, leading to lower amounts on the sampler. On the other hand, rainfall events are one of the main driving factors for compound transport from soil to surface water (Rozemeijer et al., 2010). However, the compound availability for transport is strongly influenced by its sorption to soil, while the duration between compound application to soil (via manure) and rainfall event directly determines the fraction available for transport (i.e. degradation occurs with time). In order to enhance understanding of the impact of precipitation on our findings, we have focused our analysis on the uppermost section of the catchment (outlet at location 2). This approach was taken due to the consistency of the detected compounds in this area and to prevent the influence of water originating from other parts of the catchment. Additionally, this area also has the highest number of monitoring sites (3) which provided us with a more comprehensive dataset. The daily precipitation amounts for the area during sampling periods were retrieved from the Royal Netherlands Meteorological Institute (KNMI) and are given in SM5. The total amount of precipitation in week 9–12 was approximately three times greater than in week 5–8 and almost 15 times greater than in week 1–4. This suggests that the dominant water and possibly compound inputs into the stream network occurred during the last sampling period. Flubendazole demonstrated a clear trend, with higher accumulated amounts quantified during the final sampling period at all three measuring locations (2, 5, 8). In contrast, sulfadiazine exhibited the opposite trend, with the lowest quantified amounts during the last sampling period. This inverse relationship could potentially be explained by the fact that sulfadiazine has low soil sorption to the soil, making it highly mobile and susceptible to transport to the stream network immediately after application via manure, but it rapidly degrades and does not persist long enough to be transported by subsequent rainfall events. Flubendazole, on the other hand, is more persistent in the environment and has higher soil sorption, which allows it to reside in the soil for a longer period of time (i.e., accumulate), partially allowing for degradation while also making it available for transport to the streams through succeeding rainfall events after manure application. Similar conclusions could be drawn regarding the leaching of these compounds into groundwater (Rakonjac et al., 2023). To further explore this issue, an insight into the transported water quantities (both rainfall triggered and in the streams) is necessary, but also a deeper investigation on compound behavior in soil and water, which was out of the scope in this paper.

### Discussion

In our earlier modeling study (Rakonjac et al., 2022), we prioritized the most commonly soil-applied VPs in the Netherlands, namely oxytetracycline, doxycycline, sulfadiazine, flubendazole, ivermectin, and dexamethasone. Among them, oxytetracycline, doxycycline, and ivermectin are characterized with relatively high sorption to soil (Lewis et al., 2016); hence, they are less mobile and their transport towards surface water is unlikely to occur. This has been partly confirmed with our measurements, where only the incidental occurrence of oxytetracycline was observed. Further, flubendazole also has a lower affinity for transport due to sorption (Van der Linden et al., 2017), but its persistence in the environment makes this compound more likely to reach the water bodies. Our measurements indicated its wide occurrence since it was detected at all 8 locations. According to their low sorption affinity, sulfadiazine and dexamethasone could be considered as a relatively mobile compounds (Lewis et al., 2016); hence, their presence in surface water might be expected. Our measurements have confirmed this for sulfadiazine, but dexamethasone was not detected at all. As also discussed in our earlier papers (Rakonjac et al., 2022; Rakonjac et al., 2023), dexamethasone is applied to soil in much lower concentrations compared to other VPs; therefore, it might escape environmental measurements in view of detection limits.

In a nearby region with comparable animal husbandry practices, Lahr et al. (2018) conducted a grab sampling campaign of surface water. Their study focused on 40 compounds, including antibiotics, antiparasitics, and hormones. Of these, 18 compounds overlapped with those targeted in our study. In total, 5 compounds were detected, where oxytetracycline, sulfamethazine, and progesterone were occasionally found, while for ivermectin and toltrazuril an incidental occurrence was observed. Despite the fact that sampling locations and periods were not identical as in our study, the difference in the number of identified compounds was profound (5 vs 22). Moreover, our measurements identified several compounds in Dutch surface water that were not previously reported in the comprehensive overview by Lahr et al. (2019). Among these compounds, flubendazole, florfenicol, and tilmicosine were consistently detected at all sampling locations during all sampling intervals (with the exception of tilmicosine at one location), indicating their widespread and consistent presence.

To our knowledge, this is the first study to determine the presence of QACs (disinfectants) in surface water in agricultural regions in the Netherlands. However, their occurrence in surface water is not surprising as some of those compounds have been found on a number of farms, in manure, food, and soil in the Netherlands (Buijs et al., 2019). According to the Biocidal Products Regulation (BPR, Regulation (EU) 528/2012) and Registration Dossier from the European Chemicals Agency (ECHA), these compounds have a broad range of applications, such as disinfection of the materials and surfaces associated with the housing or transportation of animals, but also for more general applications like the removal of algae and other green deposits. Therefore, they have a higher chance of being present in liquid manure and eventually reach the surface water via manure application to soil (UBA, 2017). The detected amounts of QACs were moderately steady over the catchment, except at location 1 where BAC-C12, BAC-C14, and BAC-C16 were found at relatively high rates, particularly during the last sampling period. However, since the affinity of QACs to passive samplers is questionable, the mentioned observations should be taken with a reserve and further investigation may be required.

Furthermore, it is essential to highlight that the absence of the 24 targeted compounds above the LOQ does not necessarily indicate their absence in the streams. It is possible that these compounds were present but not detected due to low recovery rates (see SM2). This suggests that the proposed method may not be suitable for certain compounds, such as the group of tetracyclines, which seem to have a weak affinity for the passive sampler of our choice. Conversely, compounds like fenbendazole, ivermectin, and some sulfonamides exhibited robust binding to the passive samplers but were not detected, suggesting that they were likely absent from the streams.

## Conclusion

The similarity between the cumulative response in consecutively deployed samplers and the sampler constantly deployed in parallel, which was observed in 53% of the cases, implies that Speedisks are applicable for time integrative sampling for a number of compounds. However, also deviations from time integrative sampling were observed, probably caused by the physico-chemical properties of the compound, the environmental conditions of specific locations, or a combination of those. The advantages of passive sampling (the time integrative aspect in combination with the low LOQ) are particularly evident in the potential for detecting a wide range of compounds with passive samplers. Consequently, a number of compounds originating from animal husbandry activities were identified for the first time in Dutch surface waters. Moreover, the used set-up and sampling strategy enabled identification of possible time periods during which compound emissions have occurred, while the observed spatial patterns allowed for addressing the potential compound sources more precisely. This type of information might be very useful for spatial and temporal prioritization (e.g., location/time-specific monitoring) because it indicates that different streams show a different level of pollution during the 12-week period, which can only be seen when analyzing the samplers deployed for 4 weeks. Also, our study may be used as a first step in developing a targeted monitoring program for this type of compound. Further, this knowledge might help in defining mitigation measures to reduce the emissions of chemicals from animal husbandry. For VPs and naturally occurring hormones, an option could be reducing manure applications in source areas, while for disinfectants a closer identification of sources is required.

### Supplementary Information

Below is the link to the electronic supplementary material.

Supplementary file1 (DOCX 841 KB)

## Data Availability

All data generated or analyzed during this study are included in this published article and its supplementary information files.

## Declarations

### Disclaimer

All authors have read, understood, and have complied as applicable with the statement on “Ethical responsibilities of Authors” as found in the Instructions for Authors.
