# Supplementary material for: Surface water monitoring of chemicals associated with animal husbandry in an agricultural region in the Netherlands using passive sampling
Source: Environ Monit Assess. 2024 Jun 28;196(7):670. doi: 10.1007/s10661-024-12818-5 (PMC11213807; doi:10.1007/s10661-024-12818-5)
Supplement: Supplementary file 1 — Supplementary file1 (DOCX 841 KB) [file 10661_2024_12818_MOESM1_ESM.docx]

***Surface water monitoring of chemicals associated with animal husbandry in an agricultural region in the Netherlands using passive sampling***

Nikola Rakonjac ^a *^, Erwin Roex ^b^, Henry Beeltje ^c^

^*^ *Corresponding author:* [nikola.rakonjac@wur.nl](mailto:nikola.rakonjac@wur.nl)

*Address: Droevendaalsesteeg 3, 6708PB Wageningen, The Netherlands*

*^a^ Soil Physics and Land Management Group, Wageningen University, Wageningen, the Netherlands*

*^b^ National Institute for Public Health and the Environment (RIVM), the Netherlands*

*^c^ AQUON, the Netherlands*

Supplementary Material

SM1. Targeted compounds, quantified amounts, with LOD and LOQ values

SM2. Recovery rates

SM3. Time integrative Uptake Coefficient (TUC)

SM4. Additional details on the methods and instrument settings

SM5. Daily precipitation

SM6. Internal standards used for extraction procedure

SM1. Targeted compounds, quantified amounts, with LOD and LOQ values

*Locations 1, 6, and 2, detection technique, LOD and LOQ values*


*Locations 5, 8, 3, 4, and 7*

SM2. Recovery rates

*Tv_r_ – average recovery of the 8 recovery tests*

*S_r_ – standard deviation among the 8 recovery tests*

*vc_r_ – coefficient of variation, computed as given below.*

*U_r_ – precision, computed as given below.*

*Ue_r_ – Measurement uncertainty, computed as given below.*

*Equations:*

*vc_r_ = (**S_r_)/(Tv_r_) * 100*

*U_r_ = (Tv_r_) – 100*

*Ue_r_ = sqrt [(vc_r_)^2^ + (U_r_)^2^ + U_j_^2^]*2*

SM3. Time integrative Uptake Coefficient (TUC)

TUC values per compound and location

|  | | **TUC** | | | | | | | | Average TUC | |
| --- | --- | --- | --- | --- | --- | --- | --- | --- | --- | --- | --- |
|  | | ***Loc. 1*** | ***Loc. 2*** | ***Loc. 3*** | ***Loc. 4*** | ***Loc. 5*** | ***Loc. 6*** | ***Loc. 7*** | ***Loc. 8*** | per compound | excl.  Loc. 8 |
| estrone | | 2.86 | 2.03 | 0.83 |  | 2.15 | 1.89 | 4.50 | 7.42 | 3.10 | 2.38 |
| fipronil sulfone | |  |  |  |  |  |  | 0.55 |  | 0.55 | 0.55 |
| florfenicol | | 0.61 | 0.78 | 0.47 | 0.52 | 1.04 | 1.23 | 0.53 | 1.32 | 0.81 | 0.74 |
| flubendazole | | 1.39 | 1.06 | 0.91 | 1.53 | 1.44 | 1.63 | 1.06 | 2.53 | 1.44 | 1.29 |
| flumequine | | 1.27 | 0.92 | 0.96 | 1.05 | 1.20 | 2.18 | 1.54 | 1.52 | 1.33 | 1.30 |
| lincomycin | |  |  |  |  | 0.86 | 1.18 |  |  | 1.02 | 1.02 |
| sulfadiazine | | 0.78 | 0.90 | 0.06 | 2.18 | 1.58 | 1.77 | 0.62 | 8.00 | 1.98 | 1.13 |
| sulfamethazine | | 0.93 | 0.87 | 0.41 | 1.07 | 1.16 | 1.60 | 0.67 | 2.43 | 1.14 | 0.96 |
| sulfamethoxazole | | 0.34 |  | 0.12 |  | 0.73 | 1.40 |  | 2.28 | 0.97 | 0.65 |
| sulfamethoxypyridazine | | 0.67 | 0.7 | 0.25 | 0.69 | 1.25 | 1.61 |  | 2.26 | 1.06 | 0.86 |
| sulfapyridine | |  |  |  |  | 0.57 |  |  |  | 0.57 | 0.57 |
| tilmicosin | |  | 1.85 | 0.14 | 2.18 | 3.92 | 1.09 | 1.13 | 3.46 | 1.97 | 1.72 |
| trimethoprim | |  |  | 0.16 |  | 0.75 | 0.82 |  | 2.10 | 0.96 | 0.58 |
| BAC-C12 | | 96.70 | 3.34 | 0.89 | 2.42 | 2.14 | 4.23 | 1.86 | 6.36 | 14.74 | 15.94 |
| BAC-C14 | | 123.03 | 3.52 | 1.17 | 2.15 | 2.20 | 4.96 | 1.90 | 3.69 | 17.83 | 19.85 |
| BAC-C16 | | 106.64 |  |  |  | 1.51 |  |  |  | 54.08 | 54.08 |
| BAC-C18 | |  |  |  |  |  |  |  |  |  |  |
| DDAC-C10 | | 1.48 | 1.08 |  | 0.90 | 1.13 | 2.79 | 0.64 | 11.02 | 2.72 | 1.34 |
| Average TUC | per location | 28.06 | 1.55 | 0.53 | 1.47 | 1.48 | 2.03 | 1.36 | 4.18 |  | |
|  | excl. disinfectants | 1.11 | 1.14 | 0.43 | 1.32 | 1.39 | 1.49 | 1.32 | 3.33 |  |  |

SM4. Additional details on the methods and instrument settings

*VPs*

Analysis column: GEMINI NX C18


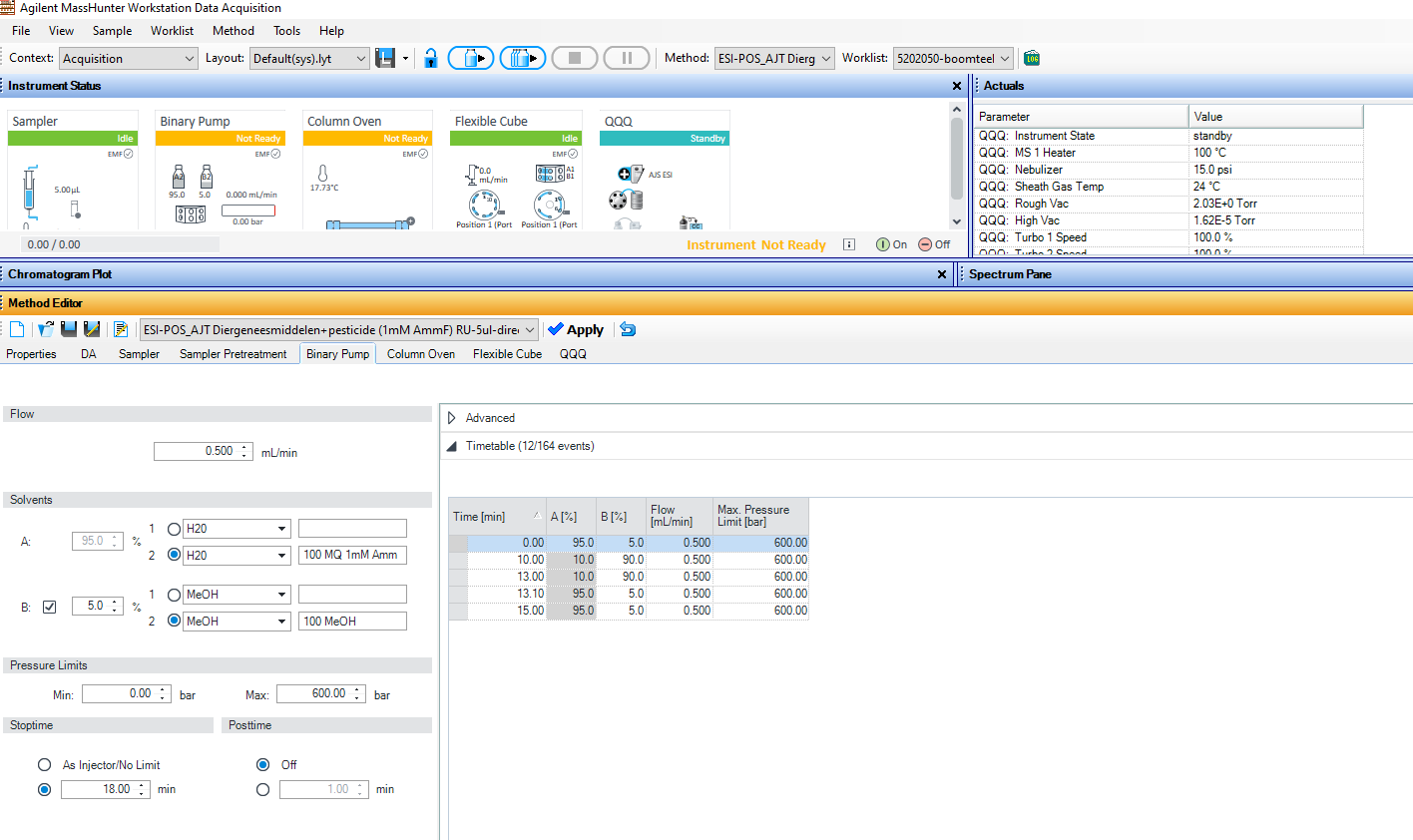


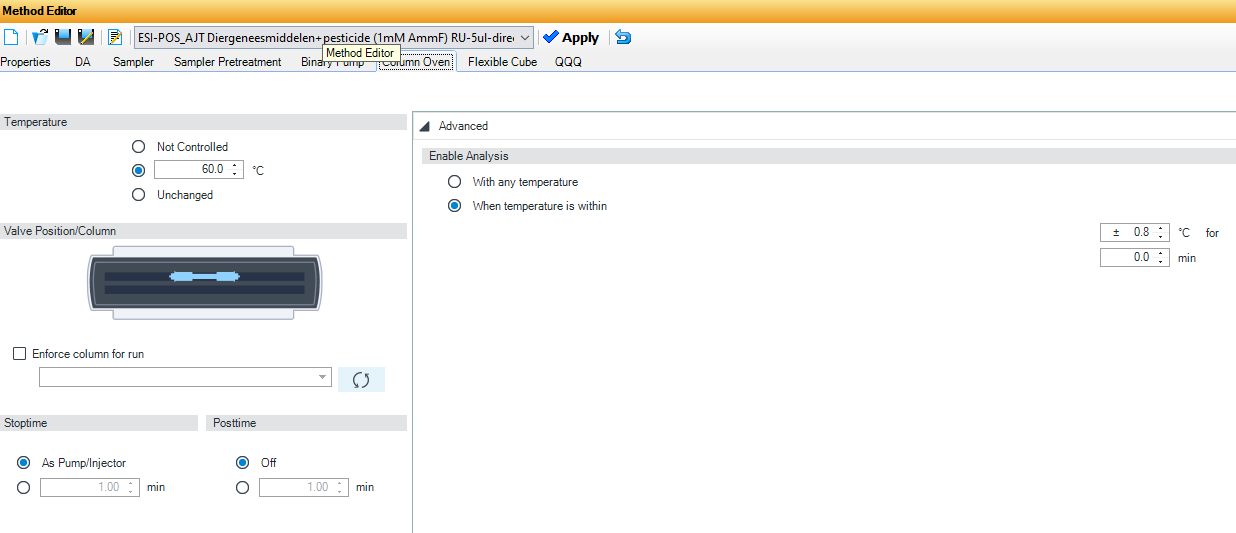


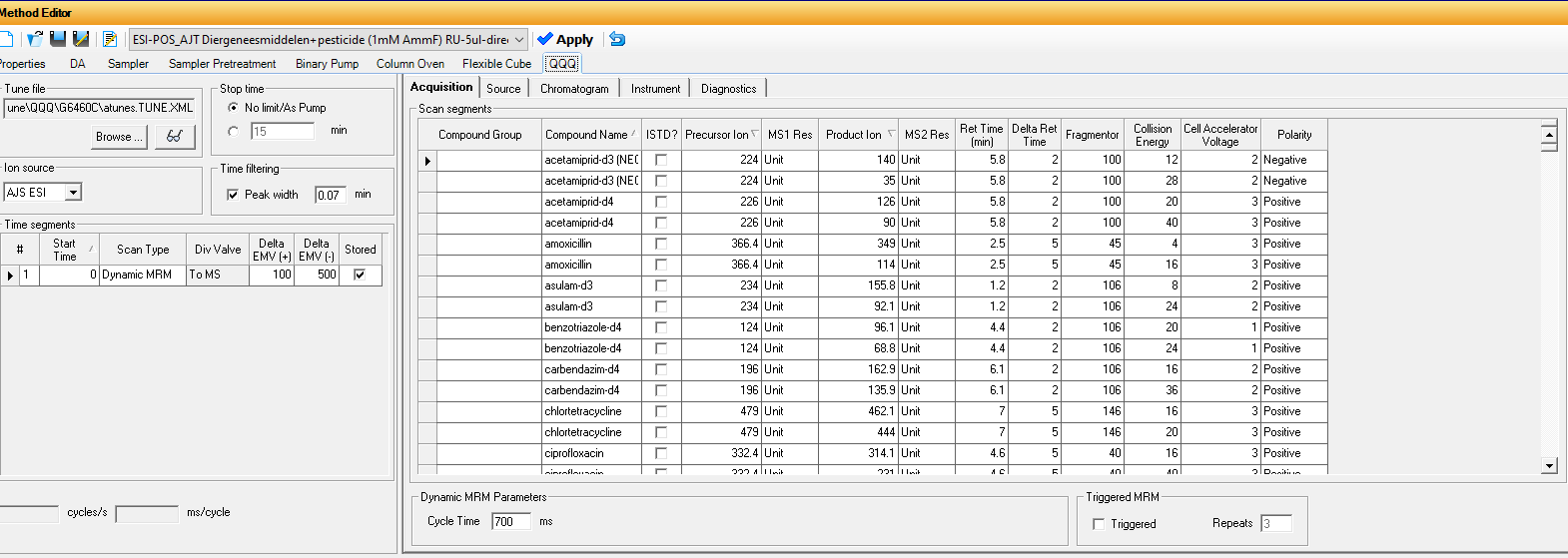


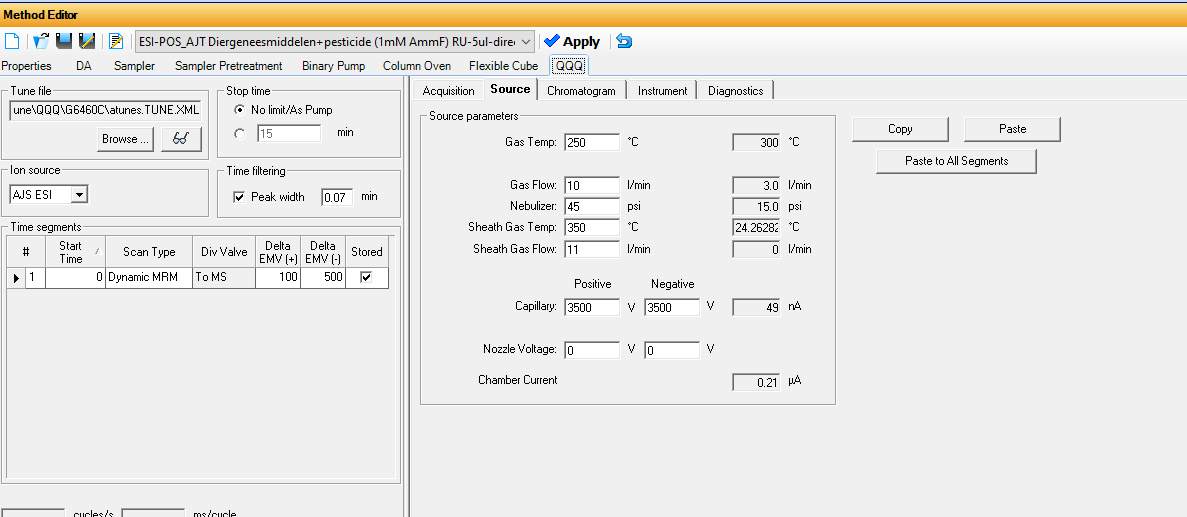

*Hormones*

Analysis column: Gemini NX C18


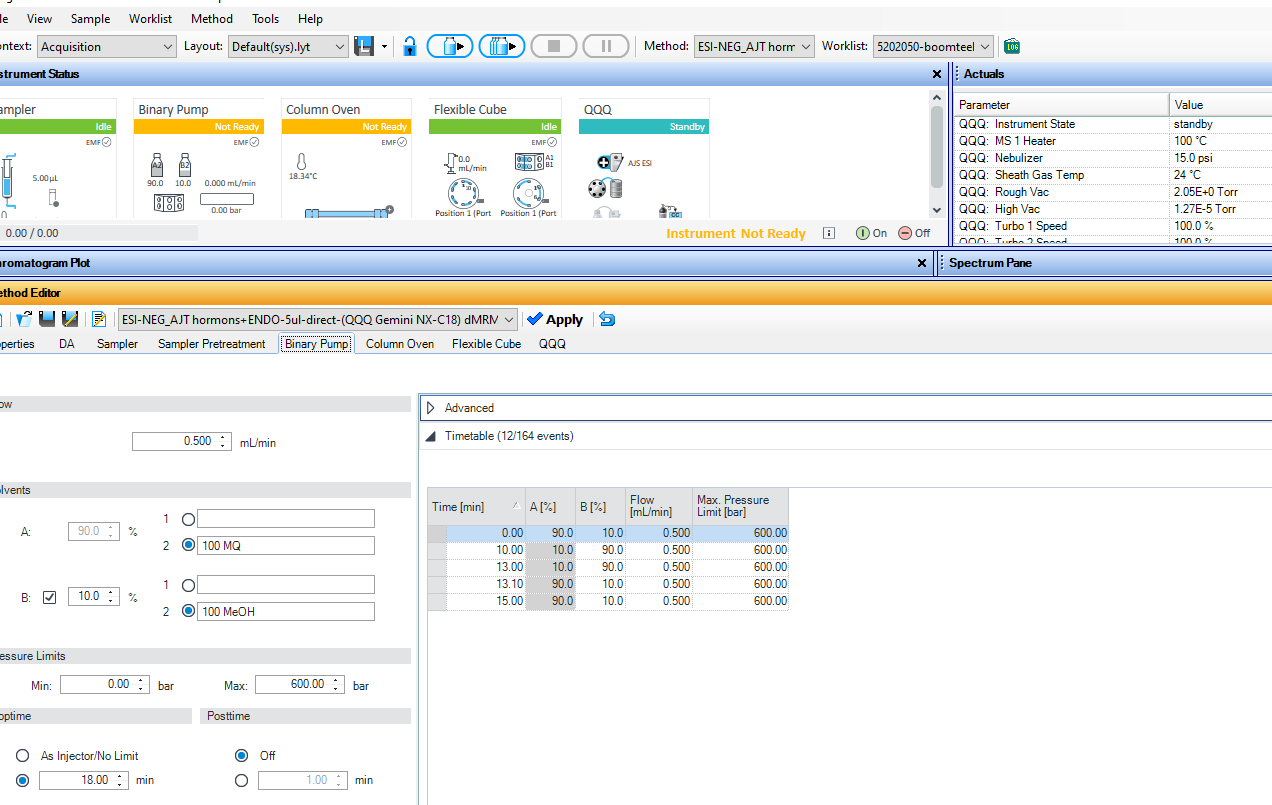


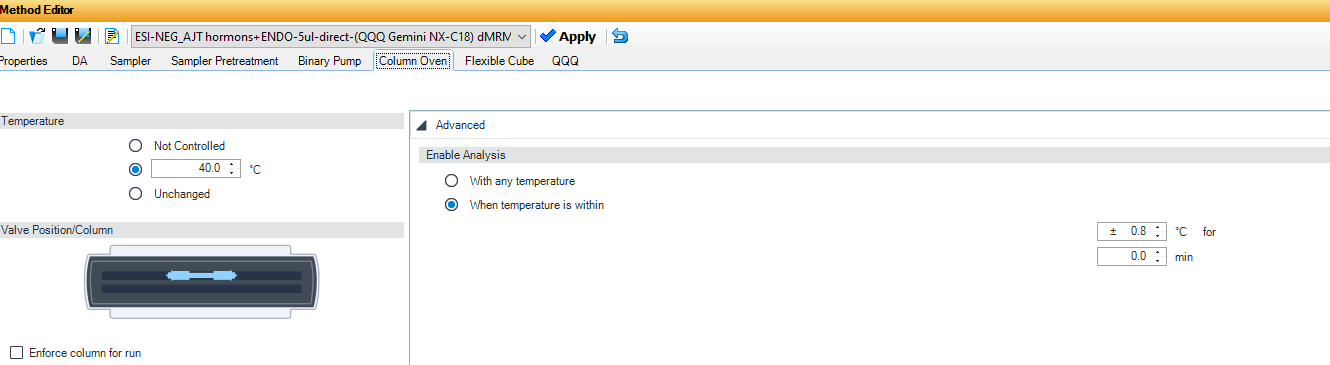


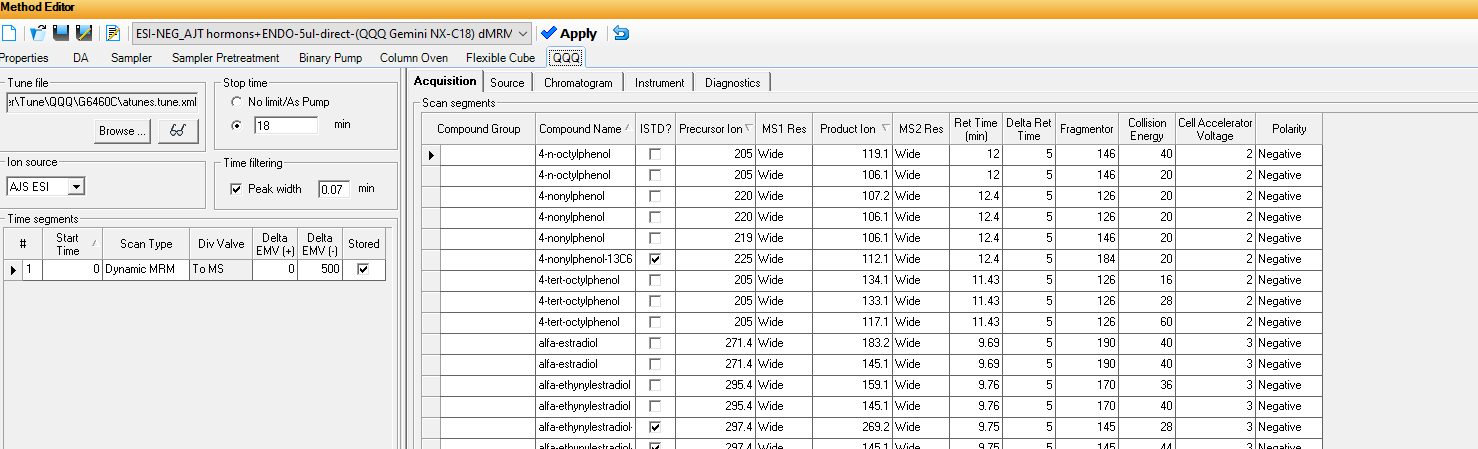


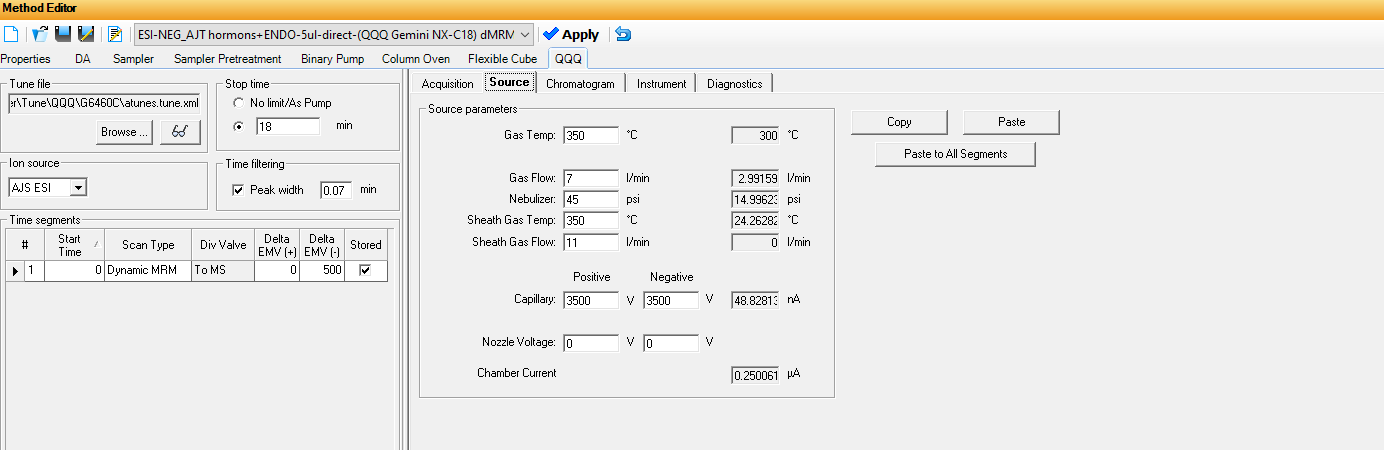

*Disinfectants*

Analysis: Kinetex Biphenyl


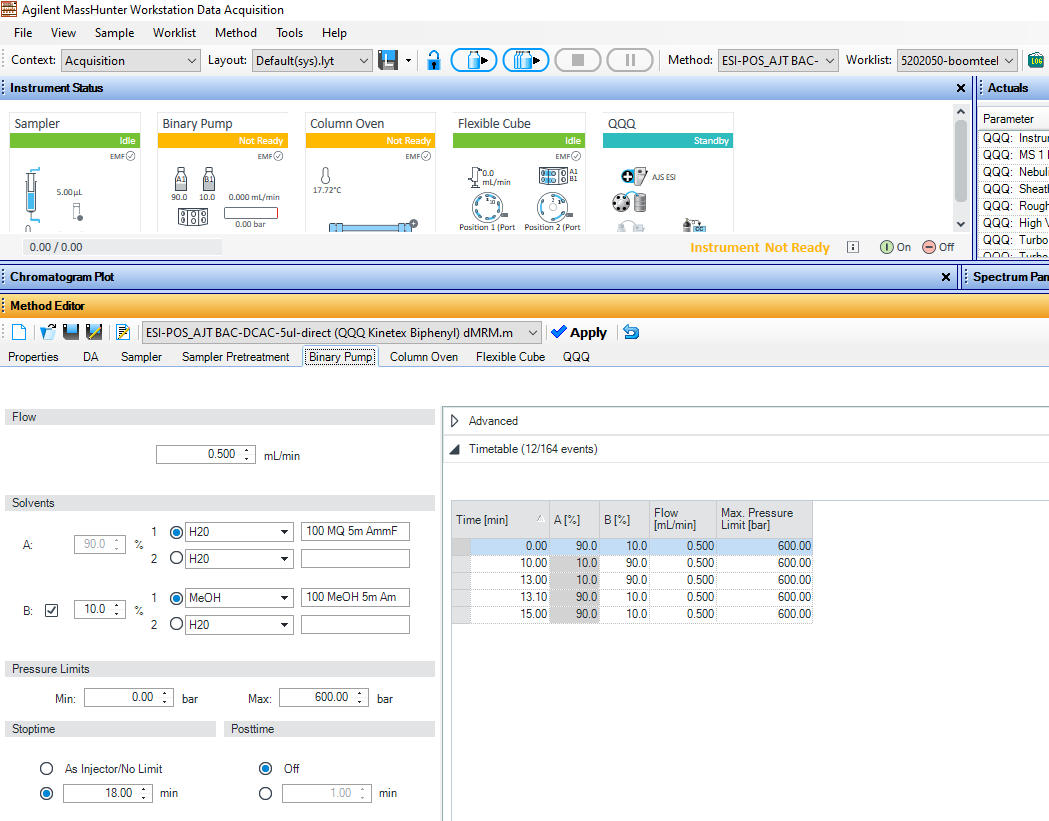


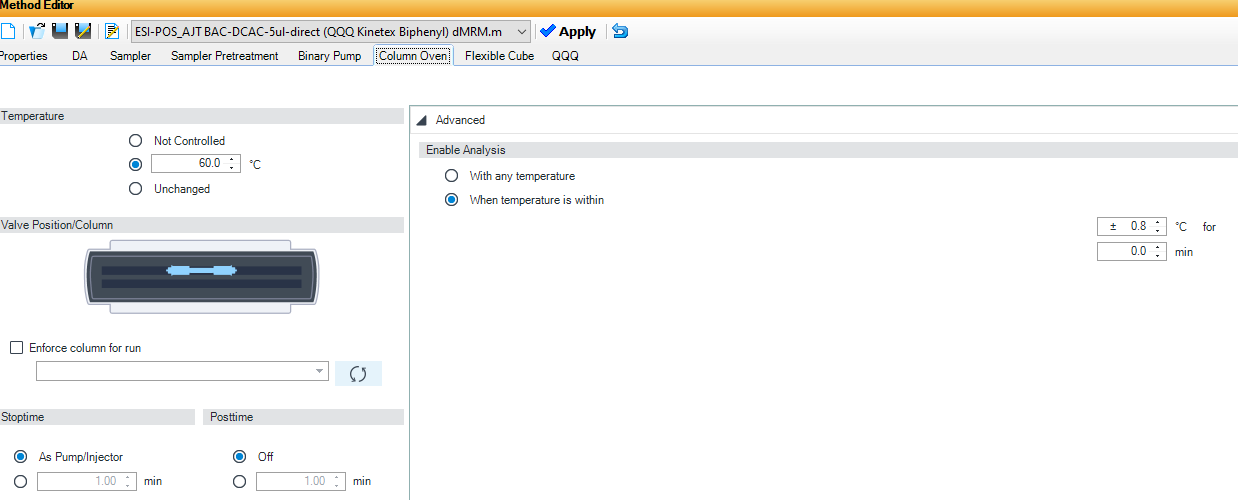


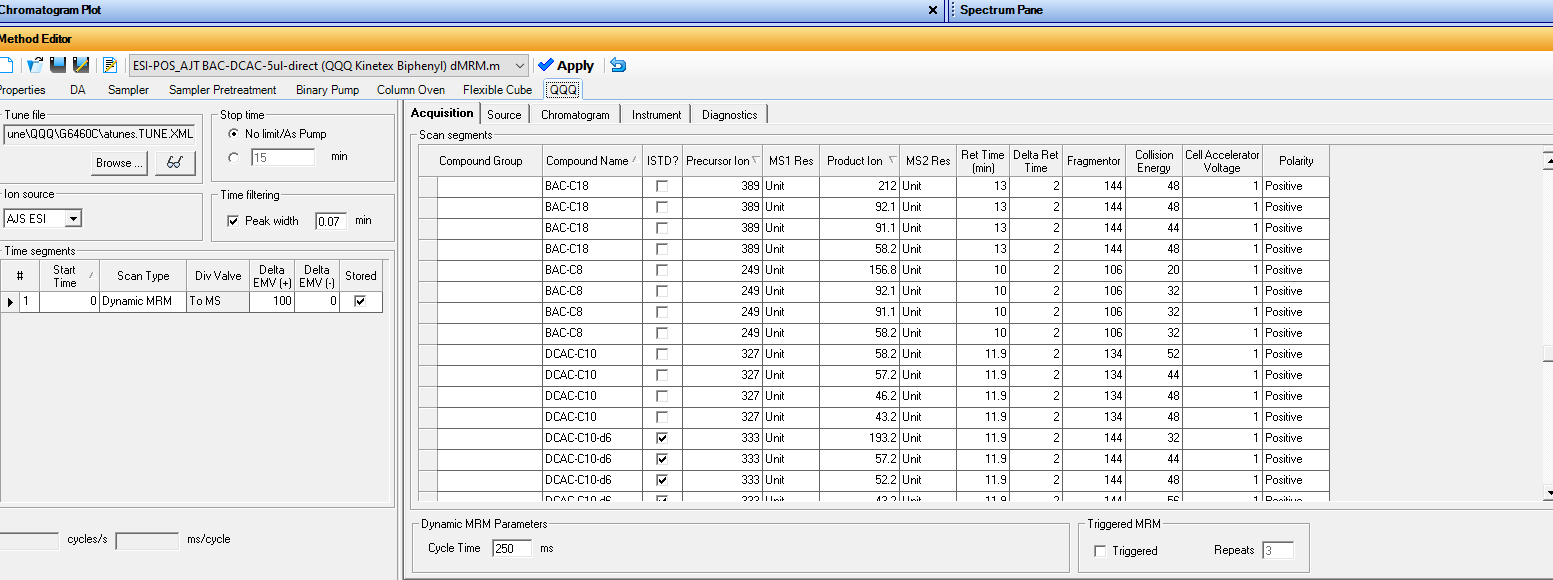


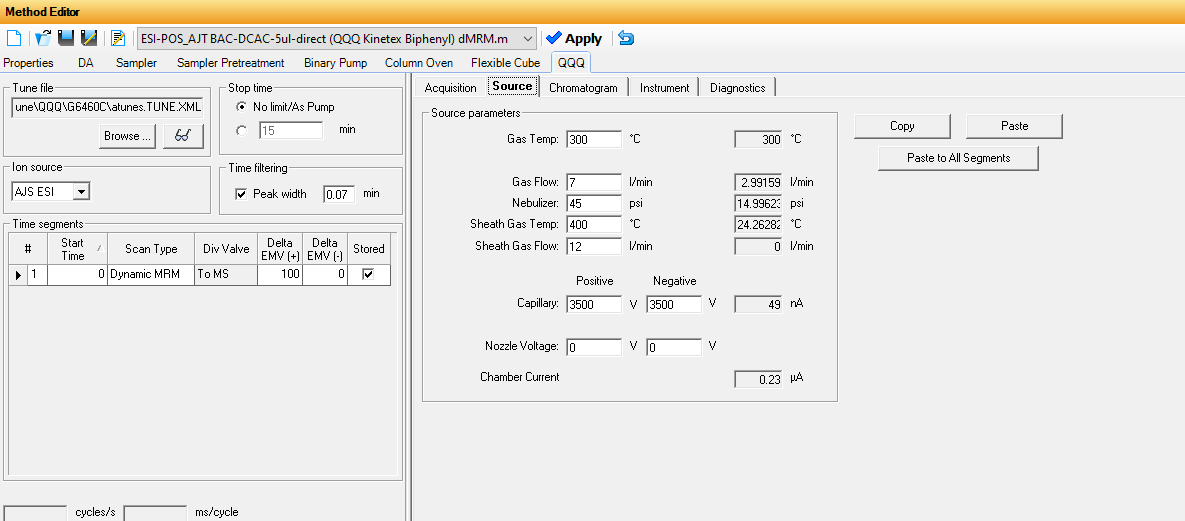

SM5. Daily precipitation

**
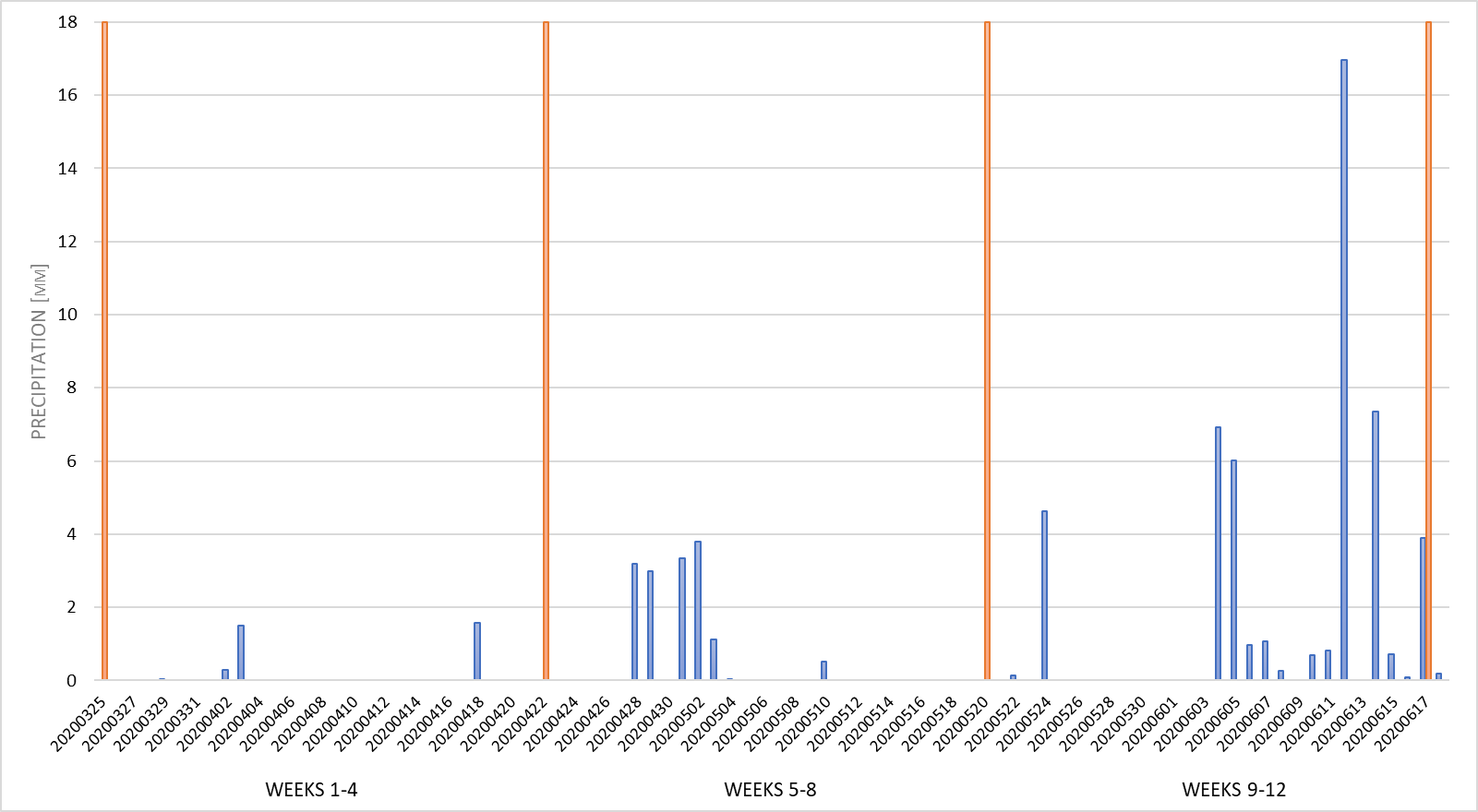
**This graph depicts the precipitation trends on a daily basis within the specified region during the sampling period. Blue bars – rainfall intensity, orange bars – days when the passive samplers were installed, changed, or collected.

SM6. Internal standards used for extraction procedure

- d6-didecyldimethylammonium iodide (HPC Standards; 674541) - disinfectants
- d7-benzyldimethyltetradecylammonium chloride (HPC Standards; 674611) - disinfectants
- benzotriazole-d4 (Sigma-Aldrich; 32566) - VPs
- erythromycin-13C2 (CIL; CLM-3672-MT-S) - VPs
- flubendazole-d3 (Sigma-Aldrich; 32839) - VPs
- naproxen-13C -d3 (CIL; CLM-7665-S) – VPs
- paracetamol-13C2 (CIL; CLM-3726-S) – VPs
- sulfadiazine-13C6 (Sigma-Aldrich; 32518) – VPs
- sulfamethoxazole-13C6 (CIL; CLM-6944-S) – VPs
- alfa-ethynylestradiol-13C2 (CIL; CLM-3375-S) - hormones
- estradiol-13C2 (CIL; CLM-803-S) - hormones
- estrone-13C2 (CIL; CLM-673-S) – hormones
